# Multiclassifier Radiomics Analysis of Ultrasound for Prediction of Extrathyroidal Extension in Papillary Thyroid Carcinoma in Children

**DOI:** 10.7150/ijms.79758

**Published:** 2023-01-22

**Authors:** Jie Li, Fantong Xia, Xiaoqing Wang, Yan Jin, Jie Yan, Xi Wei, Qiang Zhao

**Affiliations:** 1 Department of Pediatric Oncology, Tianjin Medical University Cancer Institute and Hospital, Tianjin, China.; 2 National Clinical Research Center for Cancer, Tianjin Medical University Cancer Institute and Hospital, Tianjin, China.; 3 Key Laboratory of Cancer Prevention and Therapy, Tianjin, China.; 4 Tianjin's Clinical Research Center for Cancer, Tianjin, China.; 5 Department of Diagnostic and Therapeutic Ultrasound, Tianjin, China.

**Keywords:** machine learning, papillary thyroid carcinoma, ultrasonic radiomics, children and teenagers, extrathyroidal extension, SHAP

## Abstract

**Objective:** To explore extrathyroidal extension (ETE) in children and adolescents with papillary thyroid carcinoma using a multiclassifier ultrasound radiomic model.

**Methods:** In this study, data from 164 pediatric patients with papillary thyroid cancer (PTC) were retrospectively analyzed and patients were randomly divided into a training cohort (115) and a validation cohort (49) in a 7:3 ratio. To extract radiomics features from ultrasound images of the thyroid, areas of interest (ROIs) were delineated layer by layer along the edge of the tumor contour. The feature dimension was then reduced using the correlation coefficient screening method, and 16 features with a nonzero coefficient were chosen using Lasso. Then, in the training cohort, four supervised machine learning radiomics models (k-nearest neighbor, random forest, support vector machine [SVM], and LightGBM) were developed. ROC and decision-making curves were utilized to compare model performance, which was validated using validation cohorts. In addition, the SHapley Additive exPlanations (SHAP) framework was applied to explain the optimal model.

**Results:** In the training cohort, the average area under the curve (AUC) was 0.880 (0.835-0.927), 0.873 (0.829-0.916), 0.999 (0.999-1.000), and 0.926 (0.892-0.926) for the SVM, KNN, random forest, and LightGBM, respectively. In the validation cohort, the AUC for the SVM was 0.784 (0.680-0.889), for the KNN, it was 0.720 (0.615-0.825), for the random forest, it was 0.728 (0.622-0.834), and for the LightGBM, it was 0.832 (0.742-0.921). Generally, the LightGBM model performed well in both the training and validation cohorts. From the SHAP results, original_shape_MinorAxisLength,original_shape_Maximum2DDiameterColumn, and wavelet-HHH_glszm_SmallAreaLowGrayLevelEmphasis have the most significant effect on the model.

**Conclusions:** Our combined model based on machine learning and ultrasonic radiomics demonstrate the excellent predictive ability for extrathyroidal extension (ETE) in pediatric PTC.

## Introduction

The incidence of papillary thyroid carcinoma (PTC) is 70%-90% among adult and pediatric thyroid carcinomas 1, 2. PTCs are well-differentiated, dormant tumors with low rates of recurrence and occurrence 3, 4. Nevertheless, specific histologic subtypes of PTC (high cell count, diffuse sclerosing, and infiltrative) display aggressive behavior and recurrence with extrathyroidal extension (ETE), vascular invasion, and distant metastases 3, 5, 6. According to previous research, pediatric thyroid cancer is more aggressive than adult thyroid cancer and is more susceptible to extrathyroidal extension, lymph node metastasis (LNM), and distant metastasis 6-8.

Minimal ETE is defined as a primary tumor larger than 4 cm that is contained within the thyroid gland or has invaded the surrounding strap muscles, according to the TNM classification of differentiated thyroid carcinoma by the Eighth Edition of the American Joint Committee on Cancer (AJCC) 9. In contrast, extensive ETE refers to the primary tumor invasion of subcutaneous soft tissue, trachea, larynx, esophagus, recurrent laryngeal nerve, carotid artery, prevertebral fascia, or mediastinal vessels. According to prior research, ETE is one of the independent risk factors for LNM in the central and lateral cervical areas, whether in papillary thyroid microcarcinoma or PTC 10. Furthermore, ETE is associated with higher tumor recurrence and distant metastases. Initial studies revealed that ETE is associated with a poor prognosis. The preferred treatment for papillary thyroid carcinoma in children is surgical resection, and ETE dictates the surgical technique 11, 12. Children with ETE have the option of total or partial thyroidectomy. In contrast, children without ETE have the option of lobectomy, which can retain the endocrine function of the thyroid and parathyroid glands and prevent injury to the contralateral recurrent laryngeal nerve 13. The maintenance of thyroid and parathyroid endocrine function is especially important for children and adolescents during their growth years. Therefore, it is essential to determine the presence of ETE prior to surgery.

The gold standard for determining extrathyroidal extension is surgical histopathology image analysis, but this invasive examination method cannot be used for preoperative prediction 14, 15. Ultrasound is the most prevalent method of preoperative examination for thyroid cancer. Not only is it inexpensive and noninvasive, but it is also extremely beneficial for preoperative evaluation, including for determining tumor size, extent, capsule invasion, and lymph node metastasis 16, 17. However, until recently, the majority of ultrasound examination results were based on the subjective opinion of the sonographer, making the results too dependent on the patient's medical condition and the sonographer's level of experience 16, 17. Radiomics, which is the quantitative analysis of very large quantities of data in medical images using computer technology, has been receiving a growing amount of attention as a result of its enhanced diagnostic and prognostic accuracy 18, 19.

Children and adults differently manifest thyroid tumors ultrasonographically 12. Adult-appropriate standards or models may not apply to children and adolescents. A few researchers have published reports in recent years on the ultrasonomics of papillary thyroid carcinoma in adults, but there have been no reports on papillary thyroid carcinoma in children and adolescents 20. Therefore, we developed and validated a machine-learning method based on ultrasound radiomics for the targeted prediction of ETE in papillary thyroid cancer in children.

## Materials and Methods

### Patients

**Figure 1:**
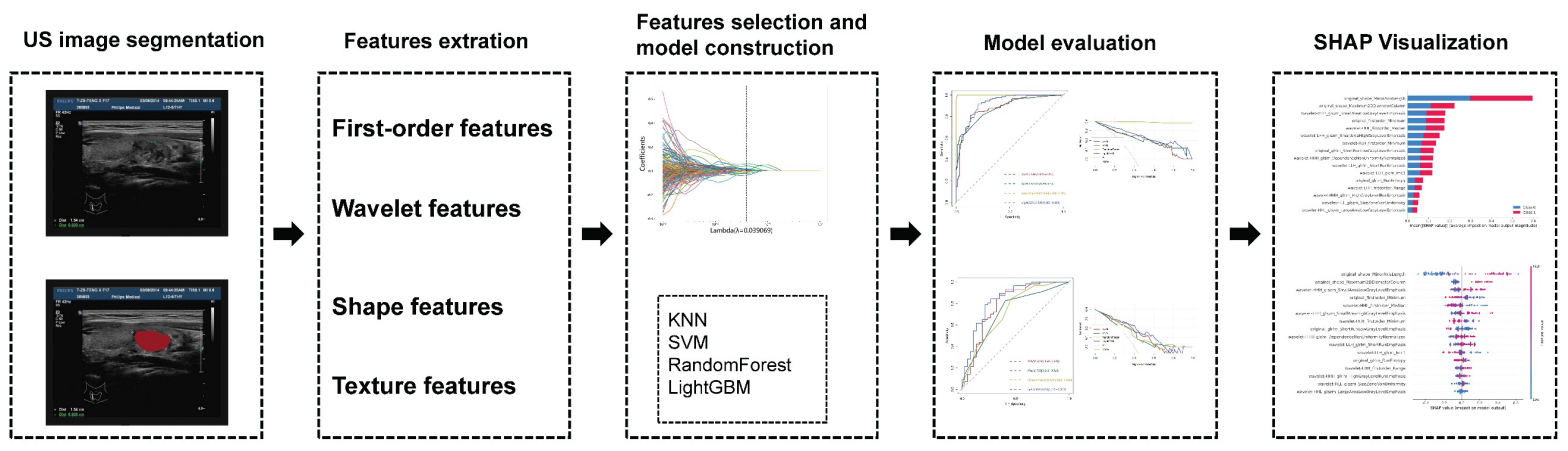
Flow chart of this study.

The study's operational flowchart is depicted in Figure 1. This study complied with the Declaration of Helsinki, and was approved by the Ethics Committee of Tianjin Cancer Institute and Hospital (No. bc2020033). The guarantees were fully informed and consented to the research. From January 2013 through August 2022, a total of 164 suitable patients were recruited from the Cancer Institute and Hospital at Tianjin Medical University. And referring to the previous study 21-25, we also divided the samples into training sets (n=115) and validation sets (n=49) in a ratio of 7:3.

The following were the criteria for inclusion: (1) patients with papillary thyroid cancer were diagnosed by postoperative pathology; (2) preoperative thyroid ultrasound examination and surgery were performed at Tianjin Medical University Cancer Hospital; (3) all patients were under 18 years of age.

The exclusion criteria were as follows: (1) patients at our institution who did not have preoperative thyroid ultrasonography; (2) patients who underwent thyroidectomy in other hospitals and only underwent cervical lymph node dissection in our hospital; (3) patients with incomplete ultrasound and pathophysiological information; (4) other types of thyroid cancer; and (5) preoperative imaging examination confirmed distant metastasis.

Age, sex, and clinical data, including tumor location and pathological features, were retrieved from medical records. This retrospective study was authorized by the Cancer Institute and Hospital of Tianjin Medical University's local ethics council, and informed consent was not necessary.

### Ultrasound Image Acquisition

All patients received a standard ultrasound evaluation before surgery utilizing Philips Q5 or Philips iU22 ultrasound equipment (both Health care, Eindhoven, the Netherlands). Modestly tilting the patient's head while they were lying supine was ideal. This allowed for a thorough assessment of the thyroid and cervical region via longitudinal, horizontal, and continuous scanning using ultrasonography. Ultrasound was used to observe the size of thyroid tumors (longest axis of nodules), tumor location (left lobe, right lobe, or isthmus), tumor location (upper, middle, and lower pole), internal echo pattern (uniform, uneven, or uneven), tumor boundary (clear, hazy, or blurred), and tumor calcification.

### Diagnostic Criteria for Image Analysis

Radiologists 1 and 2 (Radiologist 1 has 12 years of expertise in thyroid imaging, and Radiologist 2 has 10 years of experience in thyroid imaging) independently evaluated and verified the preoperative ultrasound imaging for all patients without knowledge of the histological findings. ETE can be diagnosed according to AJCC standards if any of the two following criteria is met: (1) greater than 25 percent of the lesion's circumference is in contact with the thyroid capsule or the envelope line of the lesion's contact with the thyroid gland disappears; (2) a tumor of any size exceeds the thyroid capsule and invades subcutaneous soft tissue, larynx, trachea, esophagus, recurrent laryngeal nerve, carotid artery, or mediastinal vessels.

### Region of Interest (ROI) Segmentation and Radiomic Feature Extraction and Selection

All Region of Interest (ROI) Segmentations were performed using the software ITK-SNAP (version 3.8.0, http://www.itk-snap.org) by two radiologists with more than 5 years of experience in thyroid imaging. They also had no idea whether each patient had ETE, lymph node metastasis, clinical status, or pathological status. The interclass correlation coefficient (ICC) was used to assess the feature extraction's interobserver and intraobserver agreement. An ICC greater than 0.75 was regarded as excellent 26, 27.

A total of 1,421 image features were extracted from these ROIs on ultrasound images using PyRadiomics (version 2.2.0, https://github.com/Radiomics/pyradiomics). The extracted features were divided into four categories, including shape features, first-order statistical features, texture features, and higher-order statistical features. Then, using the correlation coefficient screening method, one of the coefficients with a correlation coefficient greater than 0.90 was eliminated, leaving 217 features. Last, LASSO was used to extract the sixteen most crucial features.

### Radiomics Model Building and Model Evaluation

Four famous classifiers (KNN (k-nearest neighbor), support vector machine (SVM), random forest, and LightGBM) were used to create risk stratification models for radiological prediction after radiomics feature dimension reduction. Following that, a brief description of the four building methods mentioned previously. To begin, the k-Nearest Neighbor (KNN) classification algorithm, while theoretically mature, is also one of the most basic machine learning algorithms 28. The method works on the assumption that if the majority of the k-nearest (i.e., the closest neighbor in the feature space) samples in the vicinity of a sample belong to the same class, then the sample does as well. Second, support vector machine (SVM) is a machine learning approach that is based on statistics learning's structural risk minimization principle 29. It projected information into a multidimensional space and classified it using hyperplanes. And then, Random forest is an ensemble machine learning method for classification and regression that works by constructing a large number of decision trees and combining them into a single tree (classification) or average prediction (regression) model 30. Finally, LightGBM is a Microsoft ensemble algorithm that provides an efficient implementation of the gradient boosting algorithm. The primary advantage of LightGBM is that it dramatically accelerates the training algorithm, resulting in a more effective model in many cases 31.

During the training phase, the hyperparameters of each classifier were tuned using an iterative grid search approach to prevent overfitting and to optimize the model's performance.

Using the receiver operating characteristic (ROC) curve and calculating the area under the ROC curve, the prediction performance was evaluated (AUC). It was assessed, with the assistance of the calibration curves of the best combination model, whether the anticipated likelihood and the experimental findings were consistent with one another. This was accomplished by comparing the two sets of data. Decision curve analysis (DCA) was used to determine the clinical utility of the optimal combined model by calculating the net benefits for threshold probabilities.

### Visualization of the prediction by SHapley Additive exPlanations (SHAP)

To address the “black box” aspect of machine learning models and improve interpretability, we showed the final model using the SHapley Additive exPlanations (SHAP) dependence plot, which explains how a single feature influences the output of the LightGBM prediction model 32. This is a standard approach for interpreting machine learning model results 33. The SHAP value can be used to estimate each feature's contribution to the expected outcome. SHAP analysis assessed the SHAP value of each sample feature, which showed that feature's sensitivity to changes in model output. By linearly dividing the prediction result into the effect of each feature, the significance of features could be determined and the role of various features in the model could be displayed.

### Statistical Analysis

Continuous characteristics were analyzed using the two-sample t-test or the Mann-Whitney U test, whereas categorical characteristics were examined using the chi-square test or Fisher's exact test. R (version 4.0.0) and Python were utilized to conduct statistical analyses (version 3.6). Normally distributed data are expressed as the mean ± SD, and non-normally distributed data are presented as the mean and standard deviation, which are used to describe data that follow a normal distribution, while the median is used to describe data that do not (interquartile range). The significance value for all tests was set at p<0.05, and all tests were conducted using a two-tailed approach.

## Results

### Clinical Characteristics

With a mean age of 14.60 ± 3.52 years and a male-to-female ratio of 27:55, a total of 164 PTC patients were enrolled. A total of 103 children were pathologically identified as ETE, while 61 children were pathologically identified as non-ETE. Using stratified sampling, all patients were randomly assigned to a training group (n = 115) and a validation group (n = 49). The clinical data and sonographic characteristics of the training and validation groups are displayed in Table 1. Pathology and ultrasound image features did not differ significantly (all P > 0.05) between the two groups.

### Radiomics Features Extraction and Selection

**Figure 2:**
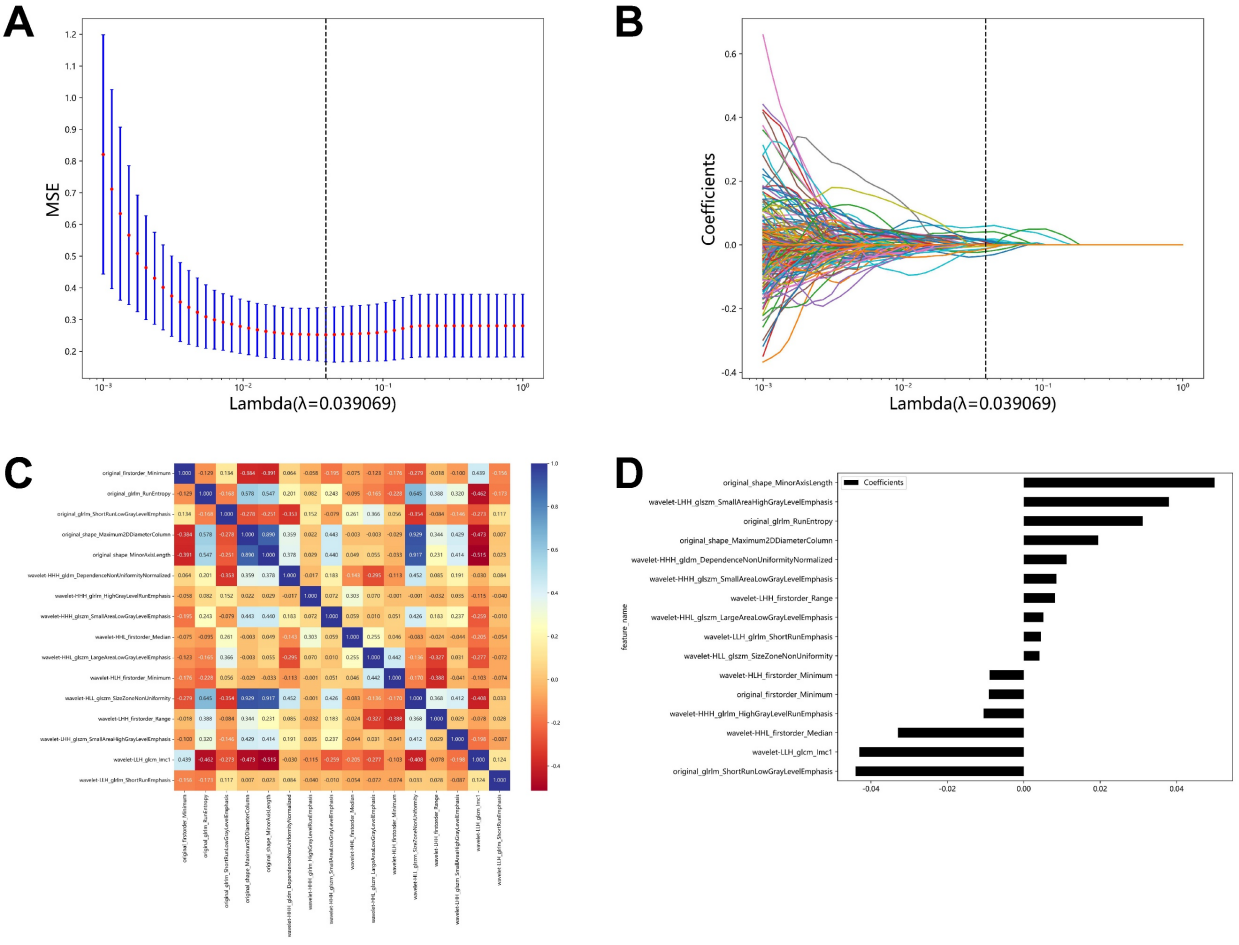
LASSO algorithm for radiomics feature selection. **(A)** Mean square error path using 10-fold cross validation. **(B)** LASSO coefficient profiles of the radiomics features. **(C)** The correlation of 16 features was extracted. **(D)** Correlation coefficients of 16 features.

For each target tumor, the ROI was manually drawn using ITK-SNAP (version 3.8.0, http://www.itk-snap.org). With intraobserver ICCs ranging from 0.802 to 0.996 and interobserver ICCs ranging from 0.799 to 0.985, favorable interobserver and intraobserver repeatability of feature extraction was achieved. And then, we extracted 1,421 image features from each grayscale ultrasound image using Pyradiomics. Then, to lower the dimension of these features, we employed the correlation coefficient screening approach and eliminated one of the features with a correlation coefficient larger than 0.90. There was a total of 217 films screened. Using LASSO regression, 16 features with nonzero coefficients were selected from the training cohort (Figure 2A-B). The 16 features are original_firstorder_Minimum, original_glrlm_RunEntropy, original_glrlm_ShortRunLowGrayLevelEmphasis, original_shape_Maximum2DDiameterColumn, original_shape_MinorAxisLength, wavelet-HHH_gldm_DependenceNonUniformityNormalized, wavelet-HHH_glrlm_HighGrayLevelRunEmphasis, wavelet-HHH_glszm_SmallAreaLowGrayLevelEmphasis, wavelet-HHL_firstorder_Median, wavelet-HHL_glszm_LargeAreaLowGrayLevelEmphasis, wavelet-HLH_firstorder_Minimum, wavelet-HLL_glszm_SizeZoneNonUniformity, wavelet-LHH_firstorder_Range, wavelet-LHH_glszm_SmallAreaHighGrayLevelEmphasis, wavelet-LLH_glcm_Imc1 and wavelet-LLH_glrlm_ShortRunEmphasis. Original_shape_MinorAxisLength had the strongest correlation coefficient with envelope invasion in papillary thyroid cancer in children (Figure 2D). We conducted correlation analyses on these 16 screened features and found that they were all relatively independent factors (Figure 2C). As indicated in Figure 2D, the correlation coefficients of these 16 characteristics were found to be highly connected with ETE in papillary thyroid cancer in children.

### Predictive Performance of Models

**Figure 3:**
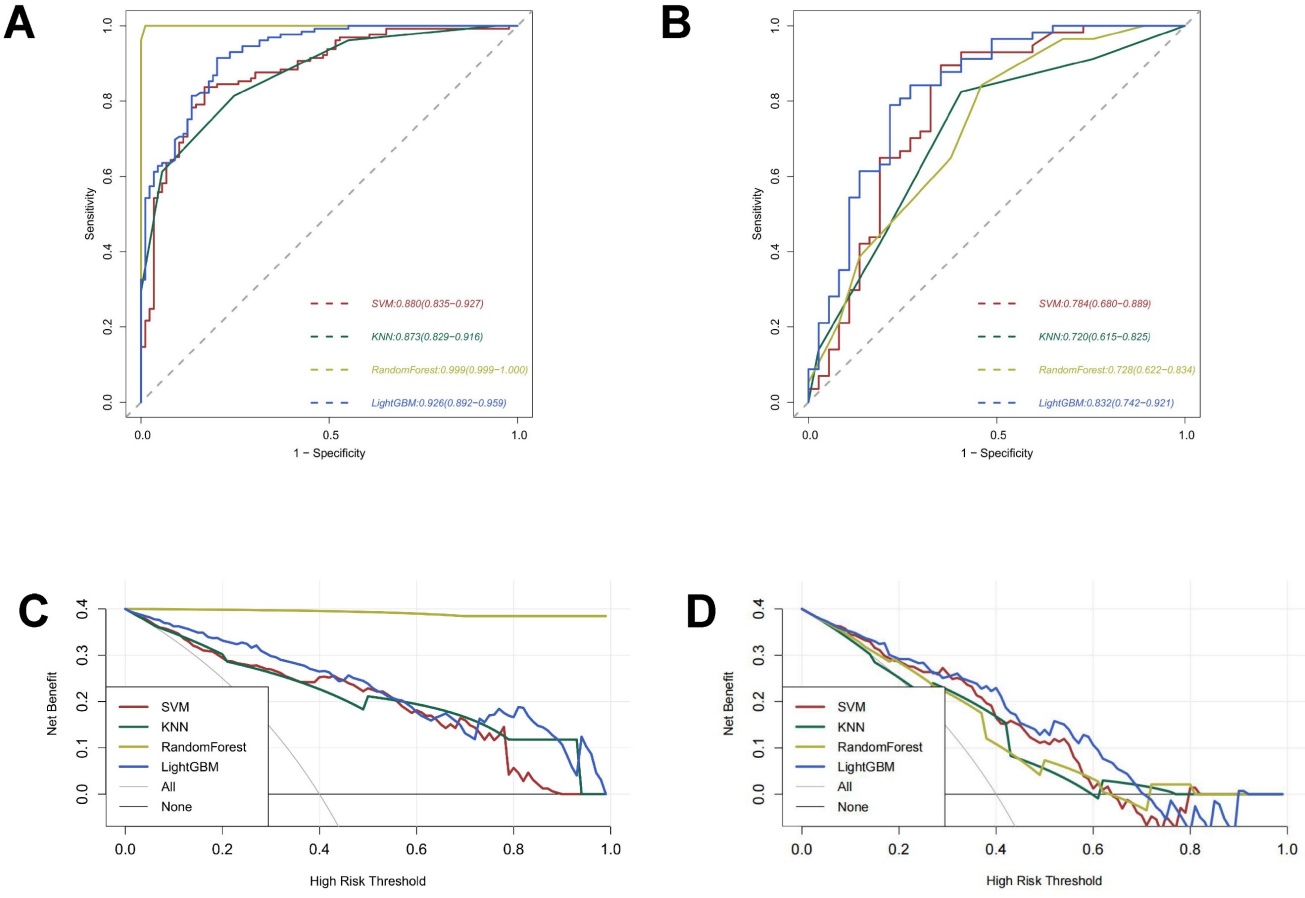
Predictive Performance of Models. **(A)** ROC curves for the radiomic models in the training cohort. **(B)** ROC curves for the radiomic models in the validation cohort. **(C)** Decision curve analysis of the radiomic models in the training cohort. **(D)** Decision curve analysis of the radiomic models in the validation cohort.

With an AUC of 0.999 (95% CI: 0.999-1.000) in the training cohort, the radiomics-random forest model produced the most satisfactory results. The AUC values of the SVM, KNN, and LightGBM models in the training cohort were 0.880 (0.835-0.927), 0.873 (0.829-0.916), and 0.926 (0.892-0.959), respectively (Figure 3A). In the validation set, the LightGBM model had the highest AUC value of 0.832 (0.742-0.921). The AUC values of the SVM, KNN, and random forest models were 0.784 (0.680-0.889), 0.720 (0.615-0.825), and 0.728 (0.622-0.834), respectively (Figure 3B).

The results of decision curve analysis in the validation and training sets are consistent with their AUC values, with the LightGBM model offering the greatest overall net benefit in the validation cohort and the random forest model providing the greatest overall net benefit in the training cohort (Figure 3C-3D).

According to the initial results, although the random forest model performs best on the training set, it performs poorly on the validation set and is not stable. While the SVM model, the KNN model, and the LightGBM models have reasonably steady performance in the training set and validation set, the LightGBM model has the best AUC value and the most significant net benefit overall.

We compared the radiomics models established by various methods with the ultrasound diagnosis of clinical pathologists. In terms of accuracy, sensitivity, and specificity, we discovered that the radiomics models generated by the four methods performed significantly better than the sonographers' judgment in both the training cohort and the validation cohort (Table 2).

### Visualization of the best radiomics model by SHapley Additive exPlanations (SHAP)

**Figure 4:**
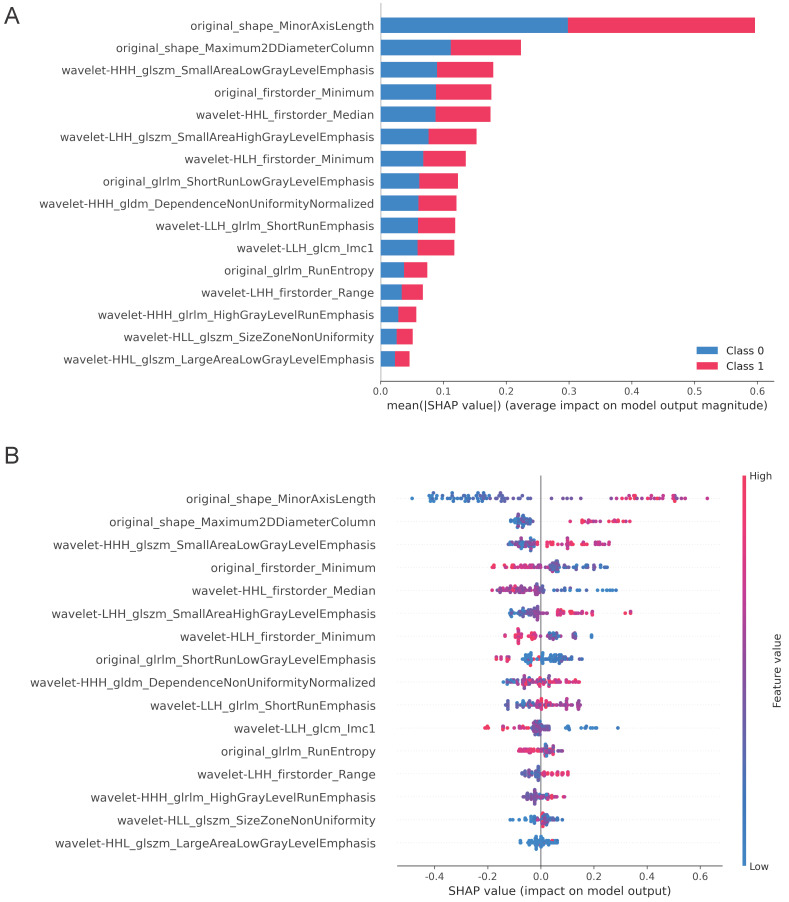
SHAP plots of the LightBGM model.** (A)** The classified bar charts of the SHAP summary plots show the influence of each parameter on the LightBGM model. Class 0: Non ETE; Class 1: ETE **(B)** The SHAP summary plot's scatter plot shows the relationship between the radiomics characteristic value and the predicted probability through colors, including positive and negative predictive effects.

In addition to accuracies and AUCs, we visualized the LightGBM model's features using SHAP analysis. The SHAP bar graph was generated by examining the mean absolute SHAP values of 16 ultrasound radiomics features to determine the degree of impact on the final projected probability (Figure 4A). Figure 4B demonstrates that each row represents a feature, the horizontal axis indicates SHAP values, and each dot represents a data sample. Different colors on the SHAP scatterplot represent the positive or negative influence of each ultrasonography sign on the projected probability. A redder hue denotes a higher value of the trait, whereas a bluer color denotes a lower value. The SHAP values for the top-ranked characteristics differed significantly depending on the presence or absence of ETE. From the results, it is clear that the differences in the three characteristics, original_shape_MinorAxisLength, original_shape_Maximum2DDiameterColumn, and wavelet-HHH_glszm_SmallAreaLowGrayLevelEmphasis, have the most significant effect on the model, and the blue dots are mainly gathered in the left portion of the axis, while the red dots mainly appear in the right portion.

## Discussion

Recurrence and mortality rates for PTC patients with ETE are higher than those without ETE 9, 15. In children and adolescents, papillary thyroid cancer is more aggressive and susceptible to capsule invasion, lymph node metastasis, and distant metastasis than in adults 2, 4, 13. Though the treatment for adults and children is similar, pediatric PTC patients with ETE must undergo total/subtotal thyroidectomy; these patients will develop chronic hypothyroidism after surgery 7, 34, 35. It is necessary to preserve as much thyroid tissue as possible to preserve the function of the thyroid gland in children during their period of growth and development. Furthermore, this technique places a higher burden on the surgeon and thus calls for a higher degree of skill; it also has the potential to hinder postoperative parathyroid function and boost the chance of recurrent laryngeal nerve injury 34, 35. A thorough diagnosis of ETE prior to surgery can, therefore, help the surgeon choose the appropriate surgical approach and limit the risk of reoperation.

Ultrasound is the preferred imaging technique for diagnosing PTC 16, 17. It can disclose the degree of PTC interaction with the neighboring thyroid capsule, but its diagnostic precision is limited. Ultrasound diagnosis of thyroid tumor capsule invasion prior to surgery is highly subjective and dependent on the diagnostic skill of the sonographer. In addition, the ultrasonographic manifestations of thyroid cancer in children and adults differ significantly. For this reason, it is essential to enhance the accuracy of ultrasound-based ETE diagnosis in pediatric PTC.

Based on US (Ultrasound) radiomics, we created four prediction models for ETE of papillary thyroid carcinoma in children 36, 37. After evaluating the four most prominent machine-learning models in radiomics, we found that the LightBGM model had excellent performance in differentiating ETE from papillary thyroid cancer in children, as well as excellent generalizability.

Each algorithm for machine learning has its own advantages and disadvantages 38-40. Without research on the subject, it is impossible to predict how well an algorithm will perform on a given machine-learning task 38-40. Almost all previous radiomics research has employed a single approach for modeling without providing any reason. The KNN (k-nearest neighbor) approach was initially suggested in 1968 by Cover and Hart. It is a mature method in principle, with simple concepts that are straightforward to comprehend and implement, and there is no need to estimate parameters. Support vector machine (SVM) learning, also known as machine learning with maximal (support) separation boundaries (vectors), is a powerful classification technology that has been applied to cancer genome classification or subtyping and is frequently employed in omics analysis 38. SVM processes nonlinear data, small samples, and high-dimensional data advantageously 38. The integrated machine learning technique known as a random forest can boost prediction accuracy without considerably increasing processing time. LightGBM (light gradient boosting machine) is a decision tree-based distributed gradient boosting system. It is distributed and efficient, having the following benefits: quicker training efficiency and reduced memory consumption, greater precision, support for parallel learning, and the ability to manage enormous volumes of data. In this study, a multiclass classification algorithm study was conducted on the same data and task, and it was discovered that KNN and SVM performed consistently, but their overall efficiency was not outstanding. Despite having the highest AUC value in the training set, the random forest model is unstable in the validation set. With the greatest AUC and best generalization in the validation group, LightGBM shows promise for additional research and validation with larger sample sets and multicenter data. In this investigation, we examined the SHAP values of our model. The mean absolute Shapley values have been developed to create an explicable radiomics model and eliminate its infamous “black box” aspect.

Our study has several limitations: (1) The algorithm for feature selection also influences the performance of the model. We did not compare the dimensionality reduction algorithms; consequently, the final feature selection may not be optimal. (2) Because of the retrospective nature of the study, case selection bias could potentially confound the results. Tumor boundaries were ill-defined in some instances with PTC. These cases were omitted from the analysis. This study was biased because the majority of the patients had PTC with ETE. (4) The radiomic model we created to distinguish ETE was created and verified at a single medical center. (5) Grayscale ultrasound images were used in our research; however, the radiomic features of multimodal ultrasound will be incorporated into the nomogram in the future. We want to use elastography and contrast-enhanced ultrasound pictures, both of which may provide more radiomic properties than standard 2D scans. (6) The sample size of this study is too small; more extensive multicenter investigations with larger samples are needed.

In conclusion, this research uses four machine-learning models of US radiomics to predict the ETE of papillary thyroid cancer in children. LightGBM-based radiomics models outperform the other three most used machine-learning algorithms in radiomics when applied to prediction accuracy.

## Figures and Tables

**Table 1:** Patient characteristics of the training and validation cohorts

Characteristic	Training cohort (%), n=115	Validation cohort (%), n=49	χ^2^	P
**Age**			3.517	0.061
<14 year	30 (26.1%)	20 (40.8%)		
≥14 year	85 (73.9%)	29 (59.2%)		
**Sex**			2.252	0.133
Female	73 (63.5%)	37 (75.5%)		
Male	42 (36.5%)	12 (24.5%)		
**Tumor size in ultrasound**			1.714	0.424
≤2cm	50 (43.5%)	19 (38.8%)		
2-4cm	47 (40.9%)	25 (51.0%)		
>4cm	18 (15.7%)	5 (10.2%)		
**Tumor location**			1.120	0.571
Left	53 (46.1%)	27 (55.1%)		
Right	59 (51.3%)	21 (42.9%)		
Isthmus	3 (2.6%)	1 (2.0%)		
**Pathological subtype**			1.744	0.418
Classic	101 (87.8%)	46 (93.9%)		
Follicular	8 (7.0%)	1 (2.0%)		
Else	6 (5.2%)	2 (4.1%)		
**Hashimoto thyroiditis**			0.225	0.635
Yes	42 (36.5%)	33 (67.3%)		
No	73 (63.5%)	16 (32.7%)		
**Tumor border**			0.594	0.441
Unclear	80 (69.6%)	37 (75.5%)		
Clear	35 (30.4%)	12 (24.5%)		
**Calcification**			0.020	0.886
Yes	88 (76.5%)	38 (77.6%)		
No	27 (23.5%)	11 (22.4%)		
**Lymph node metastasis**			0.776	0.378
Yes	102 (88.7%)	41 (83.7%)		
No	13 (11.3%)	8 (16.3%)		
**Radiological ETE**			0.024	0.876
Yes	76 (66.1%)	33 (67.3%)		
No	39 (33.9%)	16 (32.7%)		
**Pathological ETE**			0.617	0.432
Yes	70 (60.9%)	33 (67.3%)		
No	45 (39.1%)	16 (32.7%)		

**Table 2:** Summary of the performance of radiomics models and sonographer judgments in the training and validation cohorts

	Accuracy	Sensitivity	Specificity
**SVM**			
Training cohort	0.79	0.84	0.83
Validation cohort	0.72	0.89	0.65
**KNN**			
Training cohort	0.79	0.81	0.75
Validation cohort	0.73	0.82	0.61
**RandomForest**			
Training cohort	0.99	1.00	0.99
Validation cohort	0.72	0.84	0.54
**LightGBM**			
Training cohort	0.84	0.91	0.80
Validation cohort	0.78	0.79	0.78
**Radiological ETE**			
Training cohort	0.65	0.76	0.49
Validation cohort	0.63	0.73	0.44

## Abbreviations

ETE: extrathyroidal extension
LNM: lymph node metastasis
PTC: papillary thyroid cancer
ROIs: areas of interest
SVM: support vector machine
KNN: k-nearest neighbor
AUC: the average area under the curve
AJCC: the American Joint Committee on Cancer
US: Ultrasound
SHAP: SHapley Additive exPlanations
